# Needle lost in the haystack: multiple reaction monitoring fails to detect *Treponema pallidum *candidate protein biomarkers in plasma and urine samples from individuals with syphilis

**DOI:** 10.12688/f1000research.13964.2

**Published:** 2018-10-09

**Authors:** Geert A. Van Raemdonck, Kara K. Osbak, Xaveer Van Ostade, Chris R. Kenyon

**Affiliations:** 1 HIV/STI Unit, Institute of Tropical Medicine, Antwerp, 2000, Belgium; 2 Laboratory for Protein Science, Proteomics and Epigenetic Signalling (PPES) and Centre for Proteomics (CFP), University of Antwerp, Wilrijk, 2610, Belgium; 3 Division of Infectious Diseases and HIV Medicine, University of Cape Town, Cape Town, 7925, South Africa

**Keywords:** MRM, Multiple Reaction Monitoring, targeted proteomics, Treponema pallidum, syphilis, biomarker discovery, antigen test, plasma

## Abstract

**Background: **Current syphilis diagnostic strategies are lacking a sensitive manner of directly detecting *Treponema pallidum *antigens. A diagnostic test that could directly detect *T. pallidum *antigens in individuals with syphilis would be of considerable clinical utility, especially for the diagnosis of reinfections and for post-treatment serological follow-up.

**Methods: **In this study, 11 candidate *T. pallidum* biomarker proteins were chosen according to their physiochemical characteristics, *T. pallidum* specificity and predicted abundance. Thirty isotopically labelled proteotypic surrogate peptides (hPTPs) were synthesized and incorporated into a scheduled multiple reaction monitoring assay. Protein extracts from undepleted/unenriched plasma (N = 18) and urine (N = 4) samples from 18 individuals with syphilis in various clinical stages were tryptically digested, spiked with the hPTP mixture and analysed with a triple quadruple mass spectrometer.

**Results: **No endogenous PTPs corresponding to the eleven candidate biomarkers were detected in any samples analysed. To estimate the Limit of Detection (LOD) of a comparably sensitive mass spectrometer (LTQ-Orbitrap), two dilution series of rabbit cultured purified *T. pallidum *were prepared in PBS. Polyclonal anti- *T. pallidum* antibodies coupled to magnetic Dynabeads were used to enrich one sample series; no LOD improvement was found compared to the unenriched series. The estimated LOD of MS instruments is 300 *T. pallidum*/ml in PBS.

**Conclusions: **Biomarker protein detection likely failed due to the low (femtomoles/liter) predicted concentration of *T. pallidum* proteins. Alternative sample preparation strategies may improve the detectability of *T. pallidum* proteins in biofluids.

## List of abbreviations

hPTPs        Isotopically labelled proteotypic surrogate peptides

LOD          Limit of detection

MSM         Men who have sex with men

PCR          Polymerase chain reaction

TPPA        Treponema pallidum Particle Agglutination test

RPR          Rapid plasma reagin test

COG         Clusters of Orthologous Groups

FA            Formic acid

PBS          Phosphate buffered saline

NSAF       Normalized spectral abundance factor

IQR          Interquartile range

SISCAPA  Stable Isotope Standards and Capture by Anti-Peptide Antibodies

## Introduction

*Treponema pallidum* ssp. *pallidum (T. pallidum)*, a culturable ^1^ microaerophilic spirochete, is responsible for more than 8 million new cases of syphilis per year ^2^. There has been a resurgence of syphilis in a number of world regions over the last two decades ^2– 4^. In Europe ^3^ and North America ^4^, this increase has been most marked in men who have sex with men (MSM). A striking feature of these outbreaks has been the increasing proportion of cases that are occurring in patients with a previous diagnosis of syphilis ^5, 6^. Patients with reinfections are more likely to present with asymptomatic or less symptomatic disease ^5^, hence the diagnosis of reinfection is wholly dependent on subtle changes in serological tests ^7^. Two types of serological tests are used to diagnose syphilis: treponemal tests detect antibodies to *T. pallidum* and non-treponemal tests, such as the Rapid plasma reagin (RPR) test, detect agglutination secondary to the presence of anti-lipoidal antibodies reactive to material released from damaged host cells and possibly cardiolipin released from *T. pallidum* ^8^. Treponemal tests remain positive for life and are therefore of no use in the diagnosis of reinfection. Non-treponemal tests are used for syphilis post-treatment follow-up and diagnosis of reinfection. A wide range of factors can result in increases in test titers, causing syphilis to be over-diagnosed and unnecessarily treated ^7, 9– 11^. Direct *T. pallidum* detection techniques, including various nucleic acid amplification tests, have been developed, but apart from testing of primary ulcer specimens the sensitivity of these techniques is low ^12^. Even in the setting of secondary syphilis, when there is a high *T. pallidum* load in the blood ^13^, the sensitivity of polymerase chain reaction (PCR) tests reaches only 52 % on serum specimens ^12, 14^.

The *T. pallidum* genome, through evolutionary reduction, is one of the smallest of the human bacterial pathogens, with a predicted 1044 open reading frames ^15^. Approximately half of the predicted proteins have been detected through MS techniques ^16, 17^, including the semi-quantification of *T. pallidum* proteins using spectral counting ^17^. A *T. pallidum* transcriptome study demonstrated that almost all genes were expressed during peak rabbit experimental infection ^18^. This maximum utilization of the genome, well characterized proteome, and swift invasion of the organism into the bloodstream (within 24 hours after infection ^19^) make this pathogen an ideal candidate for antigen diagnostic assay development. A variety of antigen tests against other pathogens have been designed for clinical samples such as blood, cerebrospinal fluid, faeces and urine; and these have proven their utility in the diagnosis and assessment of therapeutic response in a number of infections, including *Helicobacter pylori* ^20^, *Cryptococcus neoformans* ^21^, *Cryptosporidium* ssp. ^22^, *Entamoeba histolytica* ^23^, Ebola virus ^24^ and *Mycobacteria tuberculosis* ^25^. If a highly sensitive and specific test could be developed that is able to confirm the presence or absence of *T. pallidum* in the body then this would be of considerable utility in the diagnosis of syphilis reinfections and in assessing therapeutic response. It could also be useful for the diagnosis of neuro- and congenital syphilis – two diagnoses where contemporary tests are suboptimal ^26^.

During the last decade, advanced MS-based proteomics platforms have emerged as mainstay bioanalytical tools for a broad range of clinical applications, including targeted protein identification ^27^ and bacteria identification and typing ^28^. Particularly the AQUA workflow ^29, 30^, with its use of stable isotopically labelled standard proteotypic peptides (henceforth referred to as ‘heavy’ PTPs or hPTPs) and selected/multiple reaction monitoring-mass spectrometry (SRM/MRM MS), has emerged as a powerful technique for the fast determination of multiple protein concentrations in highly complex sample matrixes such as urine (reviewed by Mermelekas *et al*. ^31^) and plasma (reviewed by Pernemalm and Lehtiö ^32^). Precise quantitation of proteins is possible by using hPTPs as internal standards that correspond to endogenous peptides created during the enzymatic digestion of the sample of interest. When combined, the endogenous and synthetic peptides elute together chromatographically and ionize with the same efficiency. Since the quantity of the labelled peptide is known, the absolute quantity of the targeted native protein can be determined by comparing MRM hPTP/endogenous peak areas. The precision and utility of this highly sensitive multiplexed method has been demonstrated on undepleted/ unenriched plasma for the detection of a panel of human cardiovascular disease ^33^ and cancer ^34^ biomarkers with a detection capability of four orders of magnitude (10 ^3^–10 ^4^ range in protein concentration) and up to femtomolar level sensitivity in plasma ^35^. Recently, a panel of 136 cancer candidate biomarkers was interrogated in unenriched urine samples using MRM, revealing detection limits of up to 25 picogram/ml urine ^36^.

With regards to infectious disease biomarker studies, MS-based approaches identified candidate biomarkers in urine for *Leishmania* sp. ^37^, which has led to the development of a urine capture ELISA diagnostic test ^38^. Considerable progress has also been made in *Mycobacterium tuberculosis* ^39– 41^ biomarker studies; recent advancements include the detection of *M. tuberculosis* in urine using IgG capture, immunodepletion and MRM methods ^42^ and MRM assay of exosomes isolated from serum samples from patients with tuberculosis ^39^.

In this study, we investigated if *T. pallidum* proteins could be detected in plasma and urine samples from individuals with syphilis using a targeted proteomics (MRM) approach. Successful development of a *T. pallidum* antigen test will most likely be contingent upon the simultaneous detection of multiple protein biomarkers to comprehensively cover different stages of disease. Eleven *T. pallidum* protein biomarkers were chosen based on a predicted specificity, high predicted abundance, and physiochemical properties. Thirty surrogate hPTPs were synthesized corresponding to eleven candidate *T. pallidum* biomarkers. Analysis of eighteen plasma and four urine samples revealed no detectable MRM signal for the endogenous peptides from the biomarkers of interest. This is likely due to the extremely low (femtomoles per liter) predicted concentration of bacterial proteins in the samples of interest, or the fact that the biomarkers are not expressed during infection. *T. pallidum* spiking experiments established a MS detection limit of 300 bacteria/ml in PBS; polyclonal anti- *T. pallidum* magnetic bead enrichment did not improve the protein detectability.

## Methods

### Study participants

Between January 2014 and August 2015, 120 patients attending the Institute of Tropical Medicine Antwerp clinic, over the age of 17 years, and in whom a new diagnosis of syphilis was made and had not received antibiotics in the preceding thirty days, were recruited into the cohort study. Thirty HIV-positive controls, in whom the diagnosis of syphilis was excluded via serological and PCR testing, were also recruited. The diagnosis and staging of syphilis was according to the Centers for Disease Control and Prevention classification ^43^, and treatment was administered according to European guidelines ^44^. All patient sera were tested for syphilis using a RPR test (BD Macro-Vue RPR card test, Becton, Dickinson and Co., Sparks, MD, United States of America (USA)) and an antibody detection *Treponema pallidum* Particle Agglutination test (SERODIA-TPPA Fujirebio Inc., Tokyo, Japan). A PCR test targeting *T. pallidum polA* was also performed on serum ^45^ and whole blood samples were tested for multiple gene targets ^46^, as previously described. Selection criteria of participants from the cohort study for the MRM assay analysis included a range of syphilis clinical stages and prioritized predicted high bacterial loads, as demonstrated by positive PCR tests and/or high RPR titres. Patients with early stage syphilis (primary, secondary, early latent) that were plasma and/or whole blood PCR positive for *T. pallidum* were expected to have the highest bacterial load ^12, 13^.

### Plasma and urine sample processing

Plasma was collected immediately before Benzathine Penicillin G intramuscular injection using 7.5 ml EDTA-coated blood collection tubes (Sarstedt Monovette, Nümbrecht, Germany). We refer to these samples as the *pre-penicillin* samples. A selection of randomly selected patients participated in an additional blood draw three hours after penicillin treatment since studies have shown penicillin to be fast acting on *T. pallidum,* leading to consequent cell lysis and antigen release ^47^. These samples are termed the *post-penicillin* samples. Plasma was chosen for the MRM assay according to HUPO guidelines ^48^. Protease inhibitors were not added to the plasma samples since previous studies did not demonstrate a significant higher protein yield with treated samples ^49^ and peptides could inadvertently be modified ^50^. Plasma were subjected to dual centrifugation in an Eppendorf 22331 centrifuge (Hamburg, Germany) in an effort to minimize cellular contamination: whole blood was centrifuged at 2000 g for 10 minutes at ambient temperature, followed by transfer of the plasma fraction to a 50 ml falcon tube and centrifugation at 2400 g for 15 minutes. All plasma were processed and aliquoted into cryovials for storage at -80 °C in a long-term freezer unit (Eppendorf U725-G Innova New Brunswick, Hamburg, Germany) until further testing. Mid-stream random-void urine samples were collected and processed following HUPO guidelines ^51^, including centrifugation for 10 minutes at 2000 g at ambient temperature in order to remove insoluble contents such as cells and casts. Urine was aliquoted into 15 ml falcon tubes and stored at -80 °C until further testing. All plasma and urine samples were processed within three hours of collection and were only subjected to one freeze thaw cycle.

### *T. pallidum* protein biomarker selection

In a previous descriptive study we used non-gel based complementary MS techniques to characterize the proteome of *in vivo* rabbit cultured *T. pallidum* ^17^. Candidate *T. pallidum* biomarker proteins for the MRM assay were chosen based on the following specific criteria: relative protein abundance (based on semi-quantitative spectral counting techniques ^17^), Clusters of Orthologous Groups (COG) functional categorization, microarray transcriptome data ^18^, protein size, physicochemical properties (i.e. previously detected by MS), predicted subcellular localization ^17^ and literature review. Each of the candidate biomarkers were digested *in silico* by subjecting the FASTA-formatted sequences to tryptic digestion, assuming 100 % digestion efficiency. Proteotypic peptides (PTPs) corresponding to these proteins were determined using ESPPredictor ^52^ and pBLAST ^53^; analysis of the protein and PTPs was performed to determine possible homology with other bacterial species and human proteins. After PTP selection was finalized, isotopically labelled synthetic peptide standards (hPTPs) corresponding to the selected PTPs were synthesized (Heavy Peptide™ *AQUA* Basic with > 95 % purity; Thermo Fisher Scientific, Ulm, Germany).

### Plasma and urine sample preparation for MRM assay analysis

Protein concentrations of urine and plasma samples were determined based on the area under curve at 214 nm using a RP-C4 column (Vydac 214TP5415; 4.6×150 mm, particle size 5 μm; Alltech Associates Inc., Lokeren, Belgium) coupled to an Alliance e2695 HPLC system equipped with a 996 PDA detector (Waters Corporation, Milford, MA, USA). For each sample, 250 µg of protein was precipitated by adding six volumes of ice cold LC-MS grade acetone (Biosolve, Valkenswaard, the Netherlands) followed by overnight incubation in freezer unit (Liebherr, Bulle, Switzerland) at -20 °C. In all cases, lo-bind Eppendorf tubes (Eppendorf, Hamburg, Germany) were used to ensure high recovery rates of proteins and peptides. Protein pellets were re-suspended in 50 mM Tris-HCl/6 M urea/5 mM dithiothreitol /10 % beta-mercaptoethanol (25 µL/100 µg protein) at pH 8.7. For the denaturation and reduction process all samples were incubated at 65 °C in a hot water bath for 1 hour. Subsequently, proteins in all fractions were diluted in 50 mM Tris-HCl/ 1 mM CaCl _2_ (75 µL/100 µg protein) and alkylated by adding 200 mM iodoacetamide (10 µL/100 µg protein) during 1 hour at ambient temperature and protected from light. Proteomics-grade modified trypsin (Promega, Madison, WI, US) was added at a 30:1 protein-to-enzyme ratio. After incubation at 37 °C in a hot water bath for 18 hours the digestion was stopped by freezing the samples. Protein digests were desalted by SPE using GracePure SPE C18-Max (50 mg) (W. R. Grace & Co., Columbia, MD, US) RP cartridges and a vacuum manifold. SPE cartridges were conditioned with 100 % methanol and equilibrated with 100 % LC/MS grade H _2_O and 0.1 % formic acid (FA). After loading the complete acidified (0.1 % FA) tryptic digest, peptides were washed with 10 % methanol and eluted with 40 % methanol/ 40 % acetonitrile (ACN) and 0.1 % FA. Eluted peptides were lyophilized and frozen at -20 °C until further analysis. Immediately before analysis, lyophilized digests were resuspended in 5 % ACN/0.1 % FA and spiked with a mixture of all hPTPs.

### MRM assay optimization and mass spectrometric analysis

Optimization of each PTP was performed on a triple quadruple mass spectrometer (Waters Xevo TQ, Waters Corporation, Milford, MA, US) in order to obtain the most intense transitions. The capillary voltage was tuned to approximately 2 kV with a source temperature of 150 °C. Desolvation temperature was set at 400 °C with a nitrogen gas flow of 800 L/h. Cone voltage, collision energy and dwell times were optimized for each of the PTPs. All PTPs were dissolved in mobile phase A (MP-A), containing 5 % ACN (LC/MS grade) and 0.1 % FA. For each of the peptides individually, the Limit of Detection (LOD) was determined by performing a dilution series in MP-A. Based on these concentrations, a mixture of all hPTPs was made. A balanced hPTP mixture has been shown to increase quantification accuracy and reproducibility compared to an equimolar mixture in previous studies ^35^. To check for possible suppressive effects of the plasma matrix, the hPTP mixture was spiked into plasma from a control study subject. A balanced mixture of hPTP (concentrations detailed in Supplementary File 1) was spiked into 50 µg of plasma digest. Chromatographic separation of the plasma and urine samples was performed on a RP-C18 UPLC column (Waters, CSH 150 × 2.1 mm, 1.7 µm at 35 °C) connected to an Acquity UPLC system (Waters Corporation, Milford, MA, USA). In order to separate all peptides as best as possible, an optimized linear gradient of Mobile Phase B (MP-B) (0.1 % FA in 100 % ACN) was applied: 5 % MP-B during 1 min and from 5 to 35 % MP-B in 5 min, followed by a steep increase to 100 % MP-B in 1 min, all at a flow rate of 300 µL/min. Based on the specific retention times of each peptide, three scheduled MRM runs of 10 minutes were generated, each of them containing 20 MS1 channels (10 endogenous ( *T. pallidum)* PTPs without isotopic label and 10 channels with a synthetic hPTP equivalent). At least three transitions (ion pairs) were selected for each peptide of interest. For each scheduled MRM analysis, 50 µg of peptides (injection loop of 5 µL) per plasma/urine sample were loaded onto the analytical column. In addition to an extensive needle wash after each injection, a blank run was performed between two subsequent clinical samples to prevent carry-over effects. Data acquisition was controlled by MassLynx version 4.1, while targeted datasets were analysed by TargetLynx, which is part of MassLynx (Waters Corporation, Milford, MA, USA). All Xevo TQ MS raw spectral files are available at PeptideAtlas ^54^ with the identifier PASS00978.

### Magnetic bead antibody-based enrichment of *T.* pallidum proteins and approximation of the MS LOD for *T. pallidum* protein detection

*T. pallidum* protein enrichment was performed using magnetic beads (Dynabeads® M-270, Life Technologies, CA, USA) coated with biotin-conjugated polyclonal *T. pallidum-* specific antibodies (PA1-73103, Thermo Fisher Scientific, CA, USA) through streptavidin-biotin conjugation. According to the manufacturer’s protocol, 10 µg of antibody was used to bind 1 mg of beads (approximately 5 × 10 ^7^ beads).

*In vivo* rabbit cultured purified *T. pallidum* DAL-1 strain extracts ^55, 56^ were kindly provided by the group of David Šmajs from the Masaryk University, Czech Republic. The original concentration of the *T. pallidum* extract was approximately 10 ^6^ bacteria/ml as quantified under darkfield microscopy using a Olympus BX41 (Olympus Corporation, Tokyo, Japan) equipped with darkfield microscope condenser DCW 1.4-1.2; magnification 10×40. Samples were stored in 1 ml phosphate buffered saline (PBS) and only subjected to one freeze-thaw cycle. Two dilution series of *T. pallidum* were prepared, each time starting in 1 ml of PBS and finally equating to eight approximate bacterial concentrations: 10 ^4^, 10 ^3^, 300, 100, 33, 10, 3 and 0 bacteria/ml.

For one dilution series, each of the eight fractions were incubated with a constant amount (~10 ^5^) of magnetic beads coated with polyclonal anti- *T. pallidum* antibodies. After incubation for two hours at 4° C and magnetic separation, the supernatant was discarded and beads were washed three times with PBS. To lyse the antibody bound bacteria, 1 ml of PBS was added to each bead sample, these were sonicated on ice using a Sonics Vibra Cell VC130 (Sonics and Materials Inc., Newtown, CT, USA) (two times 30 seconds with an amplitude of 50 %). The bead fraction was retained (retentant) after sonication by using magnetic separation. Released proteins were precipitated adding ice-cold acetone and incubated overnight at -20 °C. Tryptic digestion was performed, following the aforementioned procedure, on both the precipitated proteins (supernatant) and directly “on-bead” (retentate), to test for possible unreleased proteins during sonication. For the second dilution series (unenriched), 1 ml was directly drawn from each of the eight samples. The samples from this series were also sonicated on ice (two times 30 seconds with an amplitude of 50 %) to lyse the bacteria. Released proteins were then acetone precipitated and subsequently digested, in conformance with the other parallel series procedure.

### Liquid chromatography-electrospray ionization-LTQ-Orbitrap mass spectrometry analysis of enriched and non-enriched serially diluted *T. pallidum* samples

Peptide mixtures were separated by RPLC on a Waters nano-UPLC system using a nanoACQUITY BEH C18 Trap column (100 Å, 5 μm, 180 μm × 20 mm) connected to a nanoACQUITY BEH C18 analytical Column (130 Å, 1.7 μm, 100 μm × 100 mm) (Waters Corporation, Milford, MA, USA). Peptides were dissolved in MP-A, containing 2 % ACN and 0.1 % FA and spiked with 20 fmol [Glu1]-fibrinopeptide B, which serves as an internal calibrant. A linear gradient of MP-B (0.1 % FA in 98 % ACN) from 2 to 45 % MP-B in 45 min, followed by a steep increase to 95 % MP-B in 2 min at a flow rate of 400 nl/min. The nano-LC was coupled online with a LTQ Orbitrap Velos (Thermo Scientific, San Jose, CA, US) mass spectrometer using a PicoTip Emitter (New Objective, Woburn, MA, US) linked to a nanospray ion source. The mass spectrometer was set up in a data dependent acquisition MS/MS mode where a full scan spectrum (350–2500 m/z, resolution of 60.000) was followed by a maximum of ten CID tandem mass spectra (100 to 2000 m/z). Peptide ions were selected as the twenty most intense peaks of the MS scan. CID scans were acquired in the LTQ ion trap part of the mass spectrometer with normalized collision energy of 32 %.

Obtained spectra were screened against the *T. pallidum* reference and resequenced databases (UniProt ID proteome UP000014259 ^15^ and UP000000811 ^57^ using the MASCOT search engine (Matrix Science; version 2.1.03) based on the digestion enzyme trypsin. Carbamidomethylation of cysteines was listed as a fixed modification, while methionine oxidation was set as a variable modification. A maximum of one missed cleavage was tolerated. Mass tolerance was set to 10 ppm for the precursors and 0.8 Da for the fragment ions. False discovery rate was set at 5 %. Scaffold Q+ (version 4.6.2, Proteome Software Inc., Portland, OR, US) was used to validate MS/MS-based peptide and protein identifications. Protein identifications were accepted if they could be established at greater than 95.0 % probability according to the protein prophet algorithm ^58^.

All LTQ-Orbitrap MS/MS raw spectral data is available at PeptideAtlas ^54^ with the identifier PASS00978.

## Results

### Study subject inclusion

Eighteen syphilis-infected study participants were selected for the MRM assay analyses ( Table 1). All participants were male and identified as MSM. A third of the participants (6/18; 33 %) were HIV positive. Five (28 %) presented with primary, eleven secondary (61 %), and two early latent (11 %) stage disease. Thirteen participants were confirmed *T. pallidum-*positive by serum and/or whole blood PCR testing. Four participants had indeterminate PCR results, meaning their sample was weakly positive. A second confirmatory PCR was not performed on these samples. One patient was negative for both whole blood and serum PCR. All participants tested positive with both the RPR and TPPA tests. The median RPR value was 1/64 (Interquartile range (IQR): 1/16- 1/128). In total, 22 samples were analysed, including N = 12 pre-penicillin treatment plasma, N = 6 post-penicillin treatment plasma and N = 4 pre-penicillin treatment urine samples.

**Table 1.** Summary of the clinical and laboratory characteristics of study subjects included in this study.

Patient Number	HIV status	Syphilis stage	Sample type	Pre or post- treatment sampling ^#^	PCR Whole Blood	PCR Serum	RPR titre	TPPA titre
**1**	Positive	Secondary	Plasma	Pre	Positive	Indet.	1/512	>1/20480
**2**	Negative	Primary	Plasma	Pre	Negative	Indet.	1/4	1/160
**3**	Positive	Early latent	Plasma	Pre	Negative	Positive	1/1	1/1280
**4**	Positive	Secondary	Plasma	Pre	Positive	Positive	1/128	1/20480
**5**	Positive	Secondary	Plasma	Pre	Positive	Positive	1/128	>1/20480
**6**	Negative	Secondary	Plasma	Pre	Negative	Positive	1/128	>1/20480
**7**	Positive	Early Latent	Plasma	Pre	Positive	Positive	1/64	1/10240
**8**	Positive	Secondary	Plasma	Pre	Positive	Indet.	1/32	1/1280
**9**	Positive	Secondary	Plasma	Pre	Positive	Positive	1/512	>1/20480
			Urine					
**10**	Negative	Primary	Plasma	Pre	Positive	Indet.	1/16	1/5120
			Urine					
**11**	Positive	Secondary	Plasma	Pre	ND	Indet.	1/128	>1/20480
			Urine					
**12**	Negative	Secondary	Plasma	Pre	Positive	Negative	1/32	>1/20480
**13**	Positive	Secondary	Plasma	Post	ND	Indet.	1/128	>1/20480
**14**	Negative	Primary	Plasma	Post	Positive	Indet.	1/16	1/5120
			Urine					
**15**	Negative	Primary	Plasma	Post	Negative	Indet.	1/8	1/1280
**16**	Positive	Secondary	Plasma	Post	Positive	Negative	1/64	1/20480
**17**	Positive	Primary	Plasma	Post	Positive	Negative	1/64	>1/20480
**18**	Positive	Secondary	Plasma	Post	Negative	Negative	1/128	>1/20480

*Legend: ^#^- patients were treated with intramuscular injection with 2.4 MU Benzathine penicillin G; Indet.- indeterminate PCR result, second confirmatory PCR was not performed; ND- not done*

### *T. pallidum* protein biomarker selection

Eleven *T. pallidum* proteins were selected as candidate biomarkers ( Table 2). Most selected biomarkers had high normalized spectral abundance factor (NSAF) scores according to our previous study ^17^ (median 4.02; IQR: 1.97-6.97) and high microarray signal ratios ^18^ (median 3.05; IQR: 0.74-6.8). The median protein molecular weight was 39 kDa (IQR: 28-81). Two proteins were predicted to be located in the flagellum (TP_0249 and TP_0792), two in the ribosome (TP_0250b and TP_0244) and the subcellular localization of five proteins was unknown. Protein TP_0326, a BamA orthologue, has been experimentally shown ^59– 61^ to be localized in the outer membrane. A typical target for PCR assays is *polA*, coding protein TP_0105 ^62^. One protein, Peptidyl-prolyl cis-trans isomerase (TP_0862) was found in a previous proteomics study where it demonstrated moderate reactivity during immunoblot experiments with human and rabbit *T. pallidum* infected serum ^16^. Protein TprG (TP_0317) is part of the paralogous *tpr* gene family that encodes candidate virulence factors ^63^ and is partially homologous to Tpr E/J. According to pBLAST analysis, all chosen biomarker proteins and corresponding PTPs did not demonstrate high homology with other pathogens, non-pathogenic commensal bacterial or human proteins (data not shown). One to three corresponding well-suited PTPs were selected for each biomarker, for a total of 30 PTPs. Details pertaining to these are provided in Table 2.

**Table 2.** List of *T. pallidum* protein biomarker candidates and their corresponding proteotypic peptides (PTPs).

Number	UniProt Accession Number *	TP Number/ gene	Protein Name	Peptide Number	Peptide Sequence ^#^	Protein Weight (kDa)	Predicted Subcellular Location ^&^	COG category function	Spectral Count NSAF value in *T. pallidum* ^17^	cDNA/ DNA signal ratio ^18^
1	R9US76	TP_0105/ *polA*	DNA-directed DNA polymerase	16	TSAVSGAIP **I**ENR	112	NK	L	2.55	0.283
				17	MALNTQIQSSAADI **V**K					
				18	VHTSFVQIGT **A**TGR					
2	O83346	TP_0326/ tp92	Putative outer membrane protein assembly factor	19	TEAGGVVVQFT **I**QEGK	94	Outer Membrane	M	1.77	0.682
				20	EQWASSPGLAES **F**R					
				21	LAFANTFTSPGG **I**PK					
3	R9UVD9	TP_0249/ flaA1	Flagellar filament outer layer protein	22	LATEVGFTPSGG **A**QR	39	NK	N	7.47	16.05
				23	DESVL **I**DFAK					
4	O83834	TP_0862/ fklB	Peptidyl-prolyl cis-trans isomerase	24	GTLLDGTVFD **A**SR	28	NK	O	NF	5.29
				25	KPGVQVTSSGLQYEVV **K**					
				27	FYVPSSLGYGE **R**					
5	O83892 ^&^	TP_0922	Uncharacterized Protein	26	MPPSPC **A**VLR	33	NK	None	7.80	1.599
				30	VASVVVISVDN **R**					
				28	YFLPGEC **A**GR					
6	R9USJ3	TP_0250b/ rpsT	30S ribosomal protein S20	29	LYNGVFSSPEVV **R**	11	Ribosome	None	5.28	3.39
7	R9UU30	TP_0244/ rpsG	30S ribosomal protein S7	10	TGEEPLPV **F**TK	18	Ribosome	J	6.78	3.053
				9	ATAVGIMYDC **L**ER					
				11	LAAEILDAYHSTGT **A**FK					
8	O83337	TP_0317	Tpr protein G	1	VLDAVTAATETALQS **R**	81	NK	None	1.32	0.743
				8	GNPMSLFNLPDQQ **K**					
				2	LTGSATLEWGISYG **K** ^$^					
9	P21991	TP_0792/ flaB1	Flagellar filament core protein FlaB	6	ELSVQAANGIYS **A**EDR	31	Flagellum	N	6.97	13.82
				7	DAGDESVMNIDSPE **K**					
				12	AYIGTMTAVAMG **I**R					
10	R9UTS8	TP_0748/ cfpA/ tpn83	Cytoplasmic filament protein A	3	GVNELETHTNSL **L**R	79	Cytoplasm	S	2.75	6.79
				4	ADIGQSFASDGS **A**DQK					
				5	EYDDTDISNLPDE **R**					
11	O83417	TP_0402/ fliI	IIISP family Type III (Virulence- related) secretory pathway protein/ Flagellum-specific ATP synthase	13	EIGLASGELP **A**TR	48	Flagellum; Cytoplasm	NU	1.97	1.241
				14	SVIVSATSDESPL **A**R					
				15	VGAYQQGSDAE **L**DR					

Legend: *- UniProt proteome ID UP000014259; &- ORF was not annotated in the re-sequenced Nichols strain genome due to its length below the 150 bp limit ^15^; #- underlined/bold amino acids indicate stable isotope labelled residues; $- peptide is homologous in Tpr E/G/J protein sequences; @- subcellular location as reported in Osbak *et al.* ^17^; NK- not known; NSAF- normalized spectral abundance factor; COG- clusters of orthologous groups; COG categories: L- Replication, recombination and repair, M- Cell wall/membrane/envelope biogenesis; N- Cell motility; O- Posttranslational modification, protein turnover, chaperones; J- Translation, ribosomal structure and biogenesis; S- Function unknown; U- Intracellular trafficking, secretion, and vesicular transport.

### Multiple reaction monitoring assay optimization

**Figure 1.**
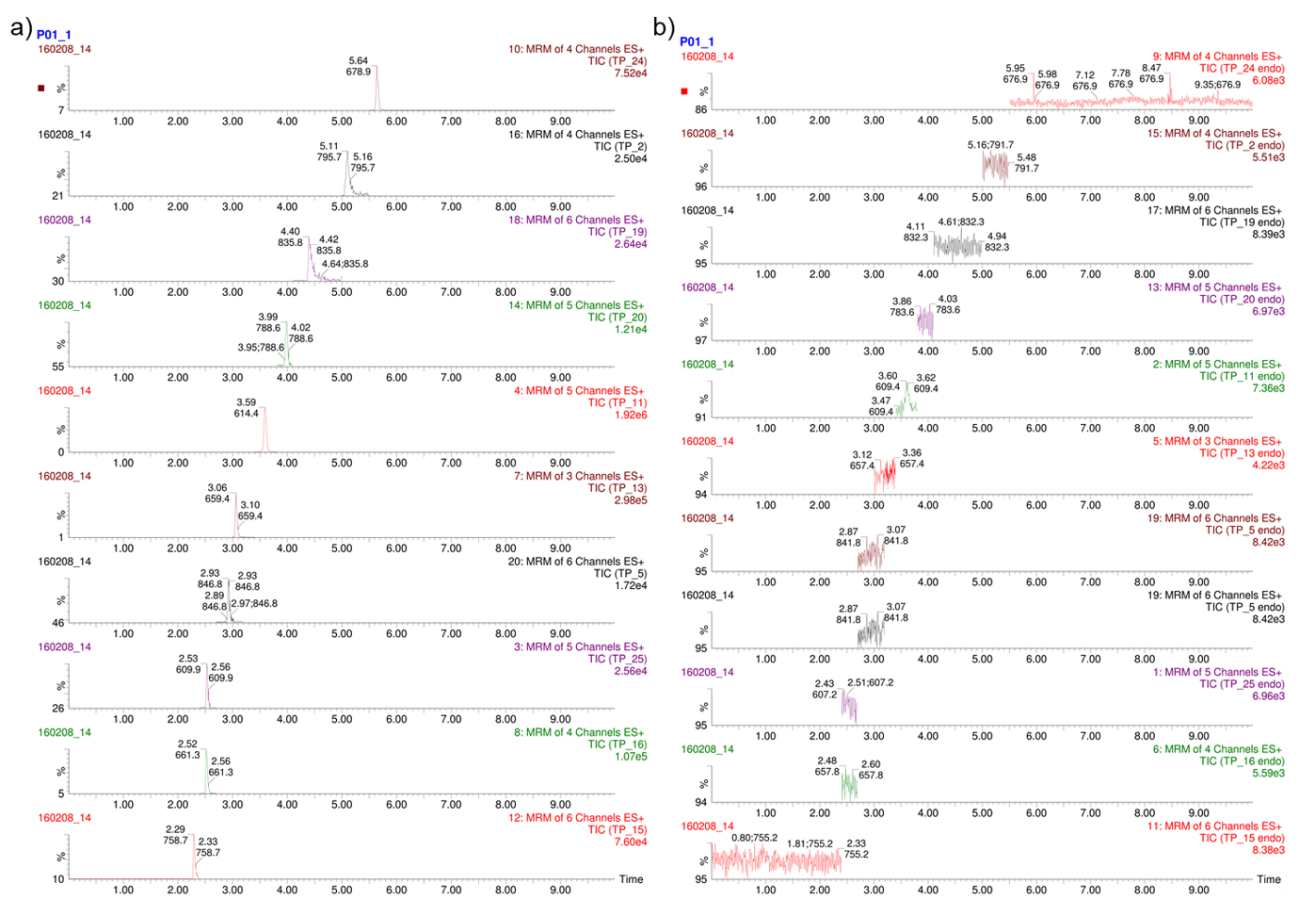
Intensity plots displaying MRM experiments on plasma from an individual with secondary stage syphilis. ( **a**) synthetic hPTPs, even numbers and ( **b**) endogenous ( *T. pallidum*) PTPs, odd numbers; gradient 1 of 3. For each peptide the number of selected transitions (channels) is reported. The x-axis shows the chromatographic retention time of the corresponding peptide while the y-axis shows the relative intensity of the MS2 signal. Note: Signal fluctuations present in the ‘endogenous’ PTP chromatogram are always the result of just one transition, often coupled with a shift in retention time and differing m/z-values differ from the hPTP run, thus these are considered to be noise.

The LOD for each peptide was determined individually by performing a dilution series of MP-A whereby the median LOD was 68.5 (IQR 14.2-176.7) picomoles. Once the peptide mixture composition was optimized based on the LOD, 2 µL of this mixture ( Supplementary File 1) was spiked into 50 µg plasma from a control patient whereby no significant variations in the signal of the hPTP transitions could be detected, indicating that there was no evidence of transition interference from the plasma. After optimizing each of the PTPs, three different sets of transitions were combined in an MRM assay based on their chromatographic retention time, as detailed in Supplementary File 1. The experiments contained a total of 141 targeted ion pairs (transitions) corresponding to 30 PTPs from eleven *T. pallidum* proteins. Ten of the eleven proteins were represented by two or more (h)PTPs ( Table 2/ Supplementary File 1). In total, three scheduled MRM assays of 10 minutes, each containing 20 peptides (10 endogenous ( *T. pallidum*) peptides and 10 hPTP standards) were developed. These assays were evaluated based on a balanced mixture of all 30 hPTPs standards. Unfortunately, although each of the 30 spiked hPTPs could be detected, none of the selected endogenous *T. pallidum* peptides could be identified in any of the MRM assays ( Figure 1; Supplementary File 2*).

### Estimation of mass spectrometry LOD and ineffective *T. pallidum* protein enrichment using magnetic bead coupled polyclonal anti- *T. pallidum* antibodies

**Figure 2.**
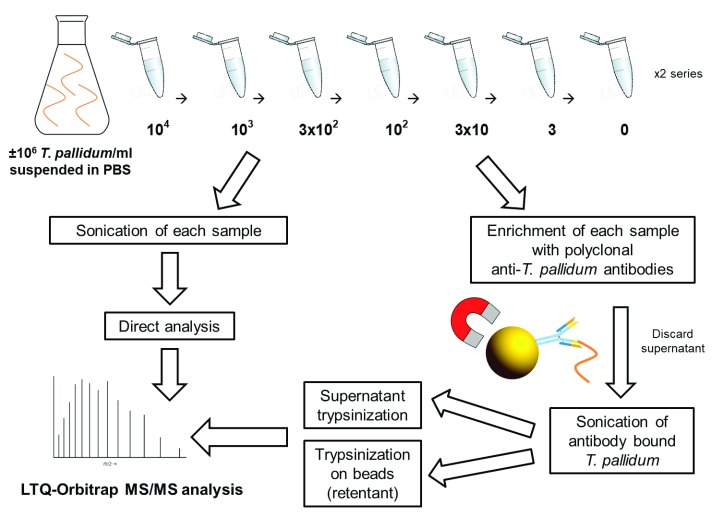
Work-flow diagram describing the estimation of *T. pallidum* protein MS LOD experiments. In total, eight different concentrations of *T. pallidum* (from 10 ^4^ to 0 bacteria/ml PBS) were treated in three different ways i) *T. pallidum* was enriched using magnetic beads coated with polyclonal anti- *T. pallidum* antibodies and lysed by sonication for release of *T. pallidum* proteins in the supernatant. Acetone precipitated proteins were trypsinized; ii) In order to detect any remaining protein on the beads, the beads were also trypsinized (retentant on-bead trypsinization); iii) As a control, non-enriched samples were sonicated and immediately trypsinized. *-proteins selected as candidate biomarkers in this study. All samples were analysed by an LTQ-Orbitrap mass spectrometer.

Two *T. pallidum* spiking dilution series were prepared in PBS and subjected to LTQ-Orbitrap MS/MS analysis in order to estimate the LOD of MS detection. One of the series was subjected to an additional polyclonal antibody coupled magnetic bead enrichment step, including sonication of the beads and subsequent separate measurement of the lysate and on-bead digestion retentate ( Figure 2).

**Figure 3.**
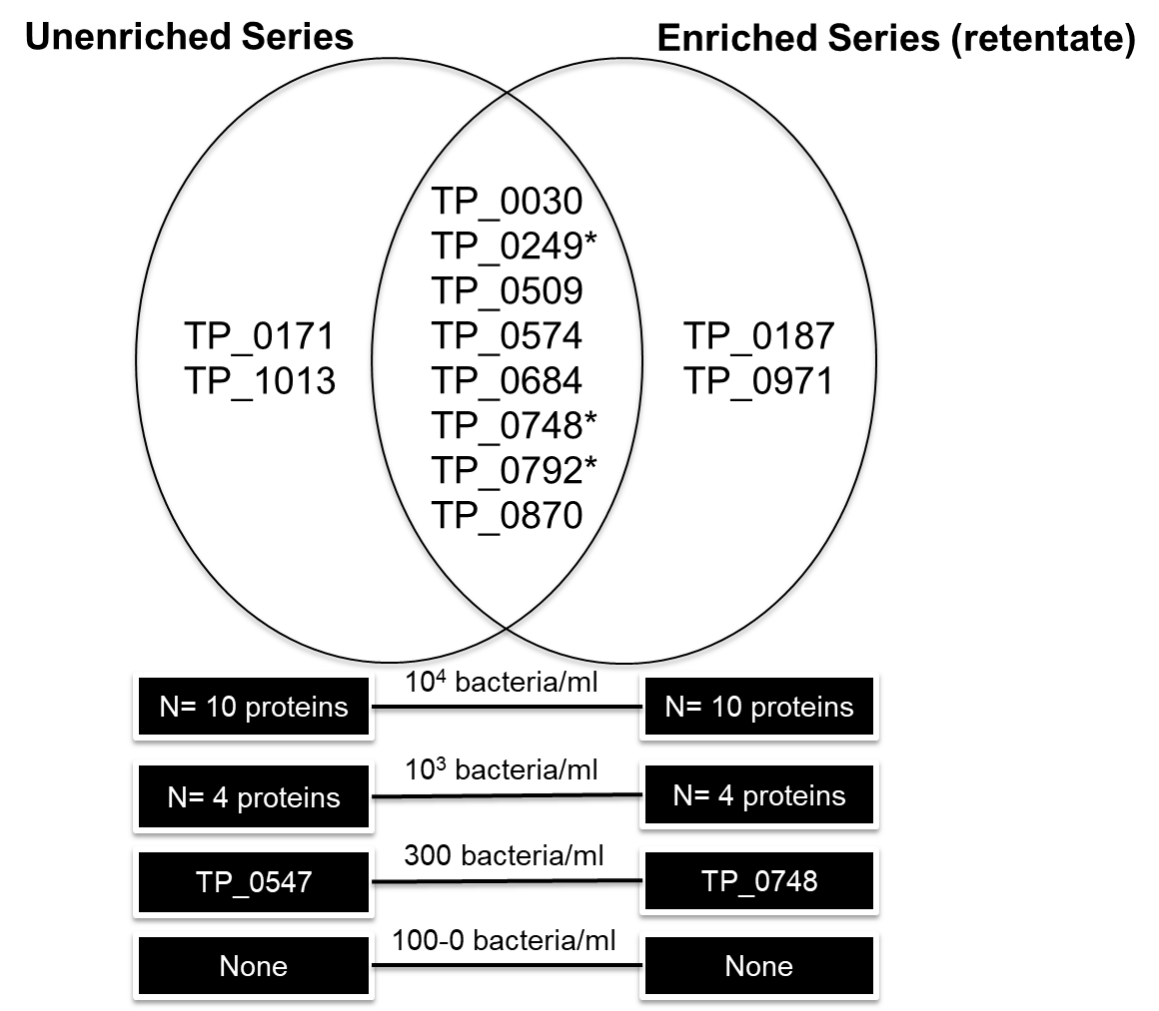
*T. pallidum* proteins detected in LOD magnetic bead coupled polyclonal anti- *T. pallidum* antibody enrichment experiments (protein and peptide identification threshold of 95 %). *-proteins selected as candidate biomarkers in this study.

Two unique *T. pallidum* proteins, Cytoplasmic filament protein A (TP_0748) and Lipoprotein antigen Tp47 (TP_0574), were found in the 300 bacteria/ml fraction in the enriched and unenriched samples, respectively ( Figure 3; Supplementary File 3). Therefore, the LOD based on a high-resolution LTQ-Orbitrap instrument was approximately 300 bacteria/ml PBS for both the antibody enriched and unenriched samples, meaning there was no significant improvement in LOD using bead enrichment. No proteins were detected in any sample concentrations for the enriched bacterial lysate (supernatant) fraction. Possibly, the sonication conditions were not harsh enough to lyse the bacteria on the beads and lysis was mainly the results of trypsin treatment under denaturing conditions. In total, eight unique *T. pallidum* proteins were found in both the unenriched and enriched retentate dilution series in one or more of the concentrations analyzed: 60 kDa chaperonin (TP_0030), Flagellar filament outer layer protein flaA1 (TP_0249), Alkyl hydroperoxide reductase (TP_0509), Lipoprotein antigen Tp47 (TP_0574), Galactose ABC superfamily ATP binding cassette transporter, binding protein (TP_0684), Cytoplasmic filament protein A (TP_0748) and the Flagellar filament core proteins flaB1/B3 (TP_0792/TP_0870). Four proteins, Lipoprotein, 15 kDa (TP_0171), 10 kDa chaperonin (TP_1013), Elongation factor Tu (TP_0187) and Tp34 lipoprotein (TP_0971) were only found in the unenriched and enriched series, respectively. Ten unique *T. pallidum* proteins were found in the highest concentration (10 ^4^ bacteria/ml) for in the enriched retentate sample (N = 10) and non-enriched sample (N = 10), two proteins detected were unique to either the enriched or unenriched samples ( Figure 3). Five unique *T. pallidum* proteins were found in the 10 ^3^ bacteria/ml sample, including N = 4 in the unenriched and N = 4 in the retentate fractions. A peptide (LSGGVAVIK) related to 60 kDa chaperonin (TP_0030) was detected in the low concentration (100/ 33/ 10/ 3 bacteria/ml) and in the negative control samples of the enriched sample series. This was likely a false-positive non-specific peptide secondary to rabbit protein contamination since this short peptide sequence is closely homologous to the *Oryctolagus cuniculus* (rabbit) 60 kDa heat shock protein, or could have originated from the beads or antibodies. As a result, it has been excluded from the analysis. Three *T. pallidum* proteins detected in both the enriched and unenriched sample series were also biomarker candidates tested in the MRM assay experiments: Flagellar filament core protein flaB2 (TP_0792), Cytoplasmic filament protein A (TP_0748) and the Flagellar filament outer layer protein flaA1 (TP_0249). Detailed information about the identified proteins, peptides, coverage and search parameters can be found in Supplementary File 3. Rough concentration calculations estimated that our target PTPs would be present in the femtomoles per liter range in human *T. pallidum* infection (calculations presented in Supplementary File 4).

## Discussion

The *T. pallidum* MRM assay designed in this study failed to detect any of the 30 targeted proteotypic peptides related to eleven candidate *T. pallidum* protein biomarkers in eighteen plasma and four urine samples from individuals with syphilis. A number of explanations are possible. The foremost is the extremely low predicted concentration of bacterial proteins compared to host proteins. To a large extent our estimates of *T. pallidum* bacterial load in blood are based on molecular studies. In one of the largest studies, Tipple *et al.* found that median copy numbers of Lipoprotein antigen Tp47 (TP_0574) DNA detectable per milliliter of whole blood was 127, 516 and 70 in primary, secondary and latent syphilis, respectively ^13^. Other studies have produced comparable results ^47, 64, 65^, with the exception of a recent study that found a median of 1.4 × 10 ^5^ *T. pallidum*/ml in whole blood from patients with secondary syphilis ^66^.

The concentration of *T. pallidum* in blood according to these PCR-based studies is lower compared to our estimated LOD in a shotgun experiment on diluted samples (300 *T. pallidum*/ml) since we would need a 500x higher concentration (same amount of proteins from 300 *T. pallidum* in 1 ml vs. 2 µl) to detect the 300 *T. pallidum*/ml (see Supplementary File 4). Despite this outcome, we were hoping to detect *T. pallidum* proteins in the plasma or urine of some syphilis patients because i) MRM measurements are generally more sensitive than shotgun experiments since scanning times are drastically reduced and ii) the amounts from Tipple *et al*. ^13^ were averages so we hypothesized that some patients (especially those with secondary syphilis) might have high *T. pallidum* levels detectable by MRM. These results could then motivate us to develop an (immuno)assay, capable of detecting the proteins even at low concentrations.

Little difference in *T. pallidum* abundance has been found between whole blood, plasma or serum ^12^. Not much is known about the persistence of *T. pallidum* in the human urinary tract and to our knowledge no studies have quantified *T. pallidum* in the urine of syphilis-infected patients. However, even if *T. pallidum* does not consistently persist in the urinary tract, bacterial proteins present in the blood could be filtered through the glomerulus, ending up in the urine either intact or as peptide fragments, depending on the size of the protein and state of proteolysis ^67^.

These considerations suggest that detection of *T. pallidum* proteins in human biofluids may not be possible without additional steps such as front-end immunoaffinity depletion ^68^, two-dimensional LC separation ^69^ and/or selective enrichment of target proteins/peptides (as reviewed by Shi *et al*. ^70^). These techniques, or combinations thereof, have allowed the detection of low abundance proteins up to the low- to sub-nanogram/ml level ^70, 71^ in clinical samples. For example, to reduce the wide dynamic range of plasma proteins, multicomponent single-step immunoaffinity depletion of high-abundant (host) proteins can allow up to a 10-20-fold enrichment of low-abundant proteins due to the depletion of 90–95 % of the total protein mass ^68^. However, of particular concern with this approach is the possibility of concomitant removal of low-abundance proteins due to protein binding to the antibodies or high-abundant proteins, as shown in a study that systematically analysed the antibody bound (high-abundant) protein fraction which found that this fraction contained 101 proteins at a high degree of confidence ^72^. *T. pallidum* has a high binding affinity for constituents of serum and host cells, including laminin ^73^, fibronectin ^74, 75^ and albumin ^76^, which may lead to unintentional depletion of targeted proteins if human protein specific immunodepletion would be applied. Furthermore, targeted mass spectrometric immunoassays (MSIA) that use surface-immobilized antibodies to affinity retrieve proteins from biological samples have proven their utility for clinical applications ^77– 79^. In our study, magnet bead coupled polyclonal anti- *T. pallidum* antibodies failed to significantly detect more *T. pallidum* proteins compared to the unenriched dilution series. Antibody effectivity is dictated by binding affinity; we used commercial antibodies that were to our knowledge not previously characterized as to their binding affinity or targeted proteins. Furthermore, it is unlikely that the polyclonal antibodies would bind a large range of proteins since few (<5 %) *T. pallidum* proteins are immunogenic ^16, 80^. The fact that *T. pallidum* can remain in ‘plain sight’ without invoking immune defences ^81^, together with the very low amount of outer membrane proteins compared to other human pathogens ^82^, also suggests that antibody enrichment of whole organisms and/or proteins would probably not be an effective strategy. Peptide-level immunoenrichment, also known as the ‘Stable Isotope Standards and Capture by Anti-Peptide Antibodies’ (SISCAPA) method developed by Anderson *et al.* ^83^ has shown considerable promise as a high-throughput, automated, highly multiplexed approach for protein biomarker quantification, with MRM application detection limits in the low picogram/ml range of protein concentration in plasma ^84^. If a selection of *T. pallidum* peptides could be definitively demonstrated to be present in plasma or urine, then this could be an attractive analytical approach with a strong potential for yielding the detection capabilities and precision needed for clinical applications.

However, apart from the low abundance in plasma or urine, other factors could explain why the *T. pallidum* proteins were not detected in our MRM experiments:

1. The LOD *T. pallidum* spiking experiments were performed in PBS buffer as opposed to a highly complex plasma or urine matrix background.

2. Variations in gene expression and structural components of proteins could also account for the lack of *T. pallidum* protein detection. Fluctuations in gene expression may explain why we did not find TprG, a protein implicated in phase variation which has been shown to be expressed at varying levels during infection due to changes in the number of guanine nucleotide repeats immediately upstream of its transcriptional start site ^85^. Heterogeneous *T. pallidum* protein sequence sites ^15, 17, 86^ could also confound rigid MRM assay detection parameters. Such heterogeneity has been shown ^17^ to be present in one candidate biomarker, TP_0922, although this variable site was not present in the PTPs incorporated in this MRM assay. Poor proteolytic cleavage can stem from structural features of the protein, different digestion kinetics and post-translational modifications. For example, phosphorylated residues within two amino acids of the point of cleavage can hinder proteolysis ^87^. Little is known about the extent of *T. pallidum* protein post-translational modification aside from a study that demonstrated glycosylation of the Flagellar core proteins (FlaBs) as reported by antibody and glycan staining techniques ^88^, however, the exact modification sites and extent of modification remain unknown. Other proteomics studies of *L. interrogans* have demonstrated likely roles for protein acetylation and methylation in virulence mechanisms ^89, 90^.

3. We only tested eleven out of more than a thousand predicted proteins in the *T. pallidum* proteome ^57^, a selection largely based on spectral counting ^17^ as an estimation of protein abundance. We cannot assume, however, that this indirect manner of quantifying *T. pallidum* protein levels in a rabbit testicle model directly recapitulates *T. pallidum* protein expression levels in plasma samples of syphilis-infected patients. One of the reasons for this is that protein expression may vary according to host and disease stage. Antigen detection during latent stage disease will be especially challenging since *T. pallidum* has been shown to sequester itself in protected niches such as eyes, hair follicles and nerves ^91^. Other *T. pallidum* proteins may be more suitable diagnostic biomarkers, given that they are reflective of the disease stages studied and that they are consistently present in the biofluids of interest. For example, Lipoprotein Tp47, which could still be identified in the most diluted *T. pallidum* sample (300 *T. pallidum*/ml) in this study, could be an interesting biomarker for future studies.

4. Various technical limitations such as a possible suboptimal chromatographic gradient length, modifiable proteotypic residues and protein degradation secondary to sample processing could have impeded biomarker detection. Other studies have reported chromatographic gradient lengths of 30 minutes or longer ^33, 34, 36, 39^, thus implementation of longer gradients could be considered in future studies in order to improve peptide resolution. In this study, chromatographic separations were performed in triple using shorter 10-minute gradients in order to optimize the sample throughput without the loss of MS sensitivity due to overlapping transition windows. Therefore, co-eluting peptides were split over different chromatographic runs since plasma protein availability was not a limiting factor. Oxidizable proteotypic residues, namely cysteine, methionine and tryptophan, can cause artifactual modifications during processing or storage resulting multiple forms of targeted peptides. With this said, the PTP selection process also requires a necessary balance between many different parameters, whereby selection of peptides containing suboptimal amino acid residues can sometimes remain the most favourable option. Ribosomal protein TP_0250b was only represented by one PTP, which may have limited detectability, thus future assays could ideally incorporate more than one peptide per protein.

5. Sample processing may have also contributed to protein degradation; therefore prompt analysis of fresh non-frozen biological specimens, if possible, is recommended. Moreover, alternative sample processing procedures, such as the use of molecular weight cut off filters to concentrate urine could improve protein detectability ^40^.

6. Lastly, only a limited amount of clinical samples were analysed, especially urine and the study was a single centre study with only MSM participants, therefore it is not generalizable. An improvement for future studies would be the incorporation of isotopically labelled (non- *T. pallidum*) reference standards, which have been shown to improve analytical precision, detect variations in instrument performance and aid in detecting chemical interferences ^92^.

Targeted MS approaches are only able to search for a limited amount of pre-selected biomarker candidates. A more comprehensive approach would be to take a step backwards to conduct broader shotgun proteomics in plasma and urine samples of individuals with syphilis. Shotgun approaches identifying *M. tuberculosis* antigens in urine have been previously successful ^40, 41^. A compelling study from Eyford *et al.* used a ‘deep-mining’ proteomics approach and were able to detect 254 *Typanosoma brucei rhodesiense* proteins in plasma from African sleeping sickness patients ^93^. Quantitative data- independent acquisition modes of MS analysis, including SWATH-MS ^94^, are also very promising avenues for clinical applications ^95, 96^.

## Conclusions

In an effort to identify promising *T. pallidum* diagnostic biomarkers, we designed a scheduled MRM assay incorporating 141 MRM ions pairs correlated to 30 PTPs/ 11 *T. pallidum* proteins. Factors such as the extremely low (femtomoles per liter) predicted *T. pallidum* protein concentration in biofluids, possible variable protein expression according to host/disease stage and potential presence of protein post-translational modifications likely contributed to the lack of signal detection for all candidate biomarkers investigated. Since the proteins targeted in this study were likely buried in the proverbial haystack of plasma proteins, alternative sample preparation and analysis strategies are warranted. With the rapidly progressing innovations of MS applications and technology, we believe clinical proteomics is far from its pinnacle of potential.

## Data availability

The datasets supporting the conclusions of this article are available in the PeptideAtlas ^54^ repository, with the identifier PASS00978, in addition to being provided within the article and its supplementary files.

## Consent and ethics approval

The prospective observational cohort study (SeTPAT ClinicalTrials.gov # NCT02059525) that provided the clinical samples used in this study was approved by the Institutional Review Board of the Institute of Tropical Medicine Antwerp and the Ethics Committee of the University of Antwerp (13/44/426), Belgium. Written informed consent for publication of the participants’ anonymized details was obtained from the participants. The *T. pallidum* ssp. *pallidum* DAL-1 strain used in this study was propagated in rabbits at the Veterinary Research Institute in Brno, Czech Republic. The handling of animals in the study was performed in accordance with the current Czech legislation (Animal Protection and Welfare Act No. 246/1992 Coll. of the Government of the Czech Republic). These specific experiments were approved by the Ethics Committee of the Veterinary Research Institute (Permit Number 20– 2014).

## Supplementary materials

**Supplementary File 1.** Table listing of optimized MRM parameters for 30 peptides targeting 11 *T. pallidum* proteins. (Supplementary File 1.xlsx).

**Supplementary File 2.** Examples of intensity plots.

**Supplementary File 3.** Table listing of protein and peptide reports for the LOD experiments using purified *T. pallidum* dilution series and ESI-LTQ-Orbitrap MS/MS analysis. (Supplementary File 3.xlsx).

**Supplementary File 4.** Calculations to estimate concentration of *T. pallidum* proteins corresponding to proteotypic peptides (PTPs) in human syphilis infections. (Supplementary file 4.xlsx).
