# Supplementary material for: Needle lost in the haystack: multiple reaction monitoring fails to detect Treponema pallidum candidate protein biomarkers in plasma and urine samples from individuals with syphilis
Source: F1000Res. 2018 Oct 9;7:336. Originally published 2018 Mar 19. [Version 2] doi: 10.12688/f1000research.13964.2 (PMC6248270; doi:10.12688/f1000research.13964.2)

Supplementary File 2. Examples of intensity plots displaying selected multiple reaction monitoring experiments for a plasma sample from a secondary stage syphilis-infected individual: 1a (synthetic hPTPs, even numbers) and 1b (endogenous (*T. pallidum*) PTPs, odd numbers). For each peptide the number of selected transitions (channels) is reported. The x-axis shows the chromatographic retention time of the corresponding peptide while the y-axis shows the relative intensity of the MS2 signal. Note: Signal fluctuations present in the 'endogenous' PTP chromatogram are always the result of just one transition, often coupled with a shift in retention time and differing m/z-values differ from the hPTP run, thus these are considered to be noise.

a) Gradient 1- endogenous (*T. pallidum*) PTP view

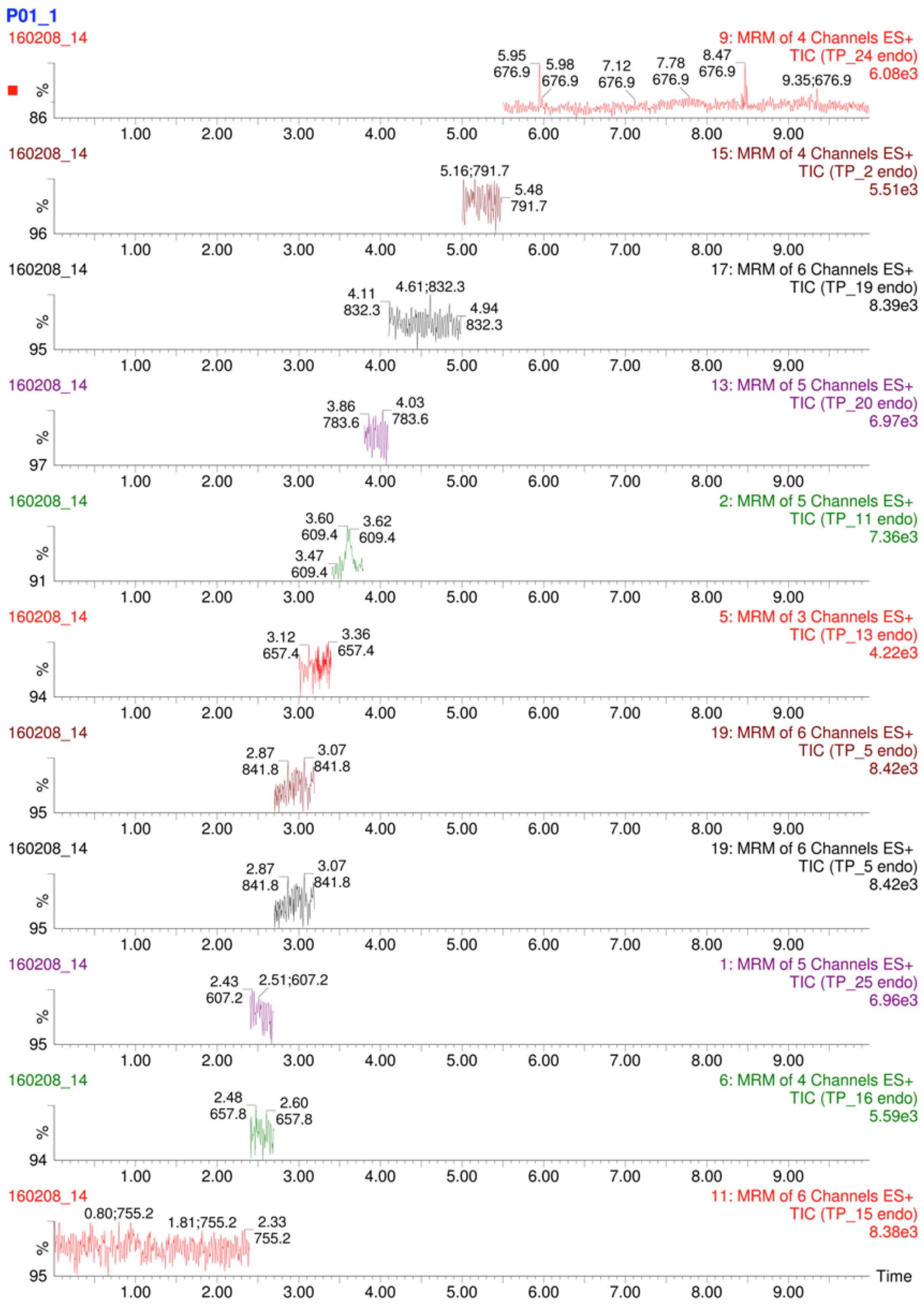

b) Gradient 1; hPTP view

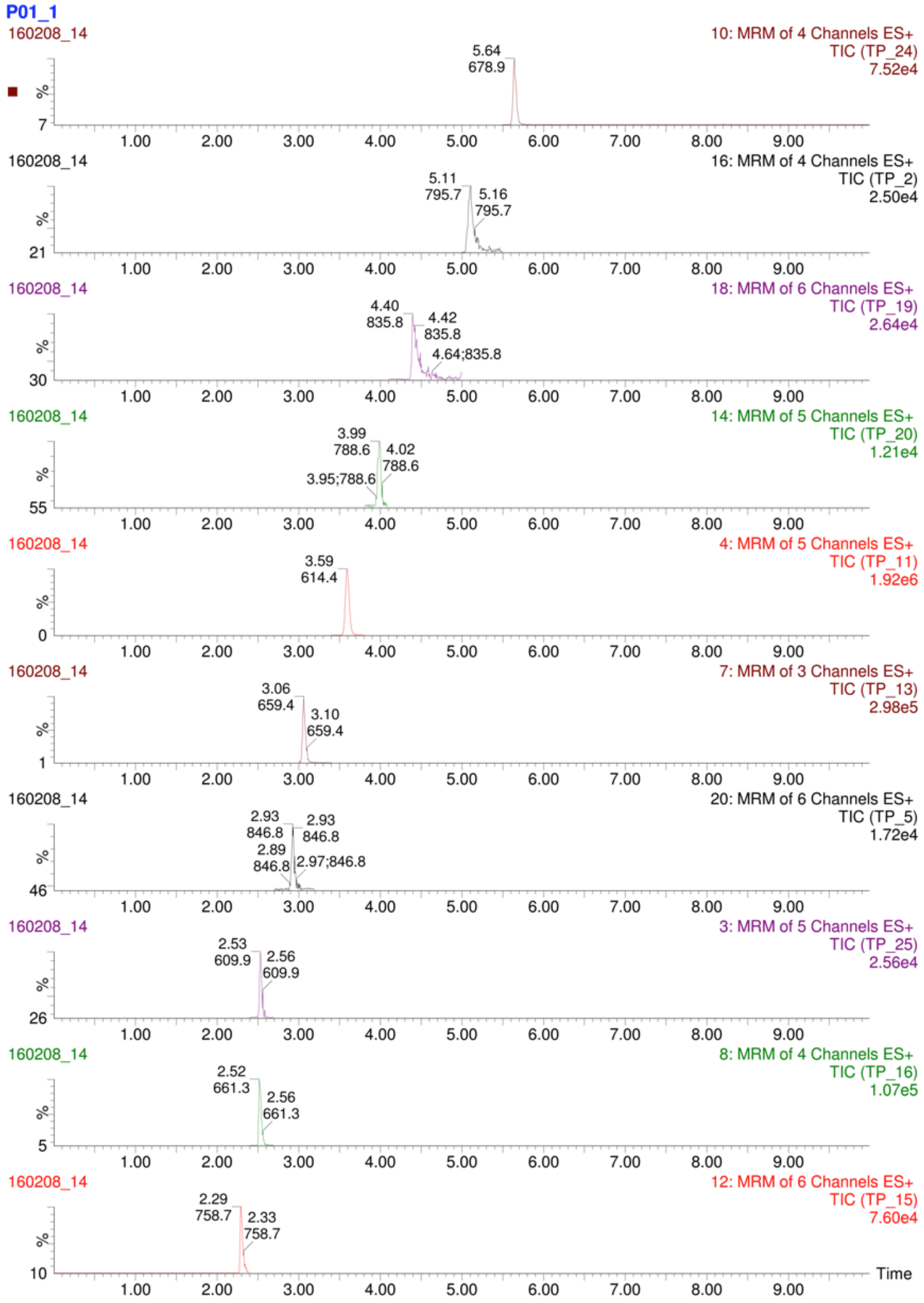

c) Gradient 2, endogenous (*T. pallidum*) PTP view

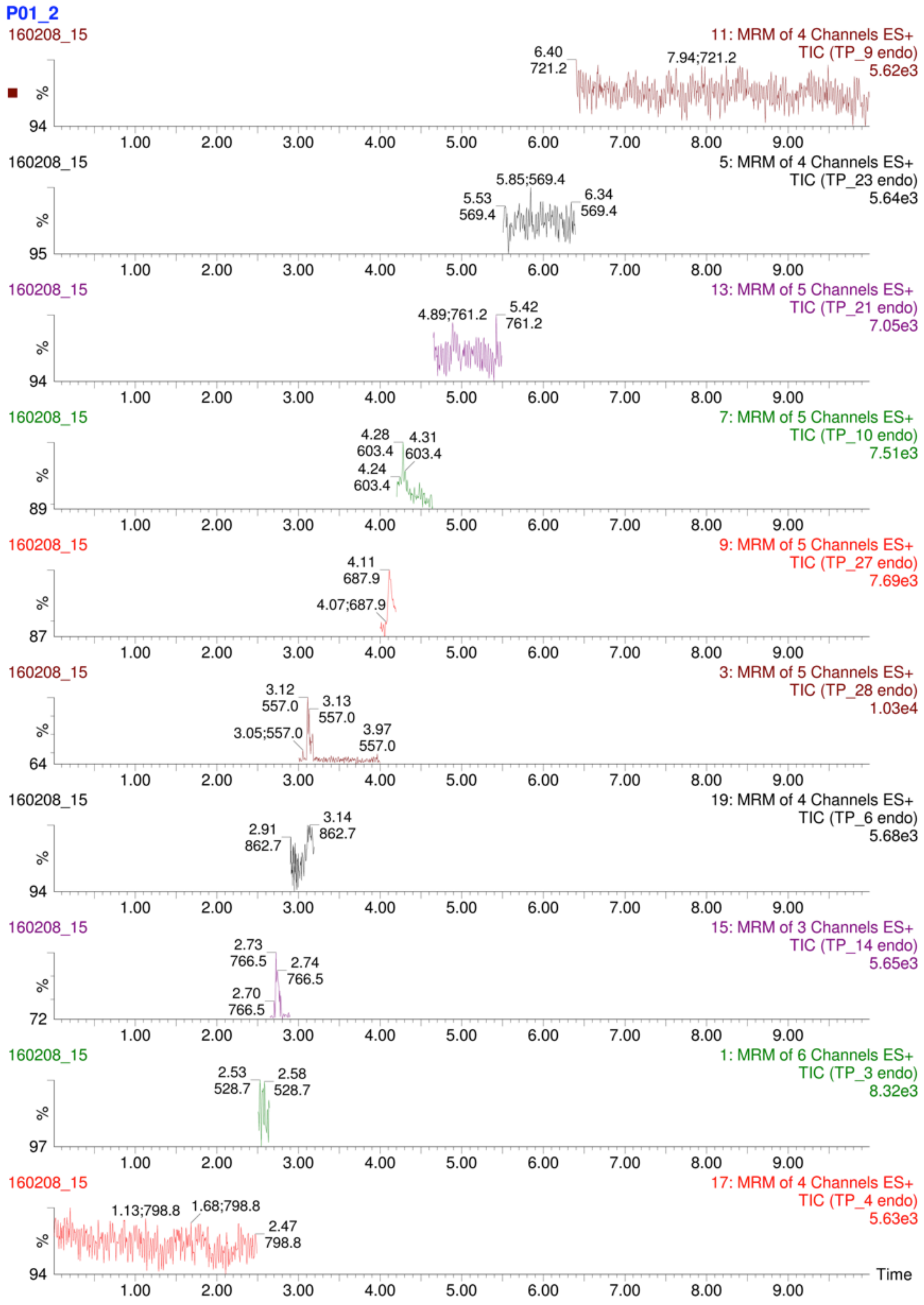

d) Gradient 2; hPTP view

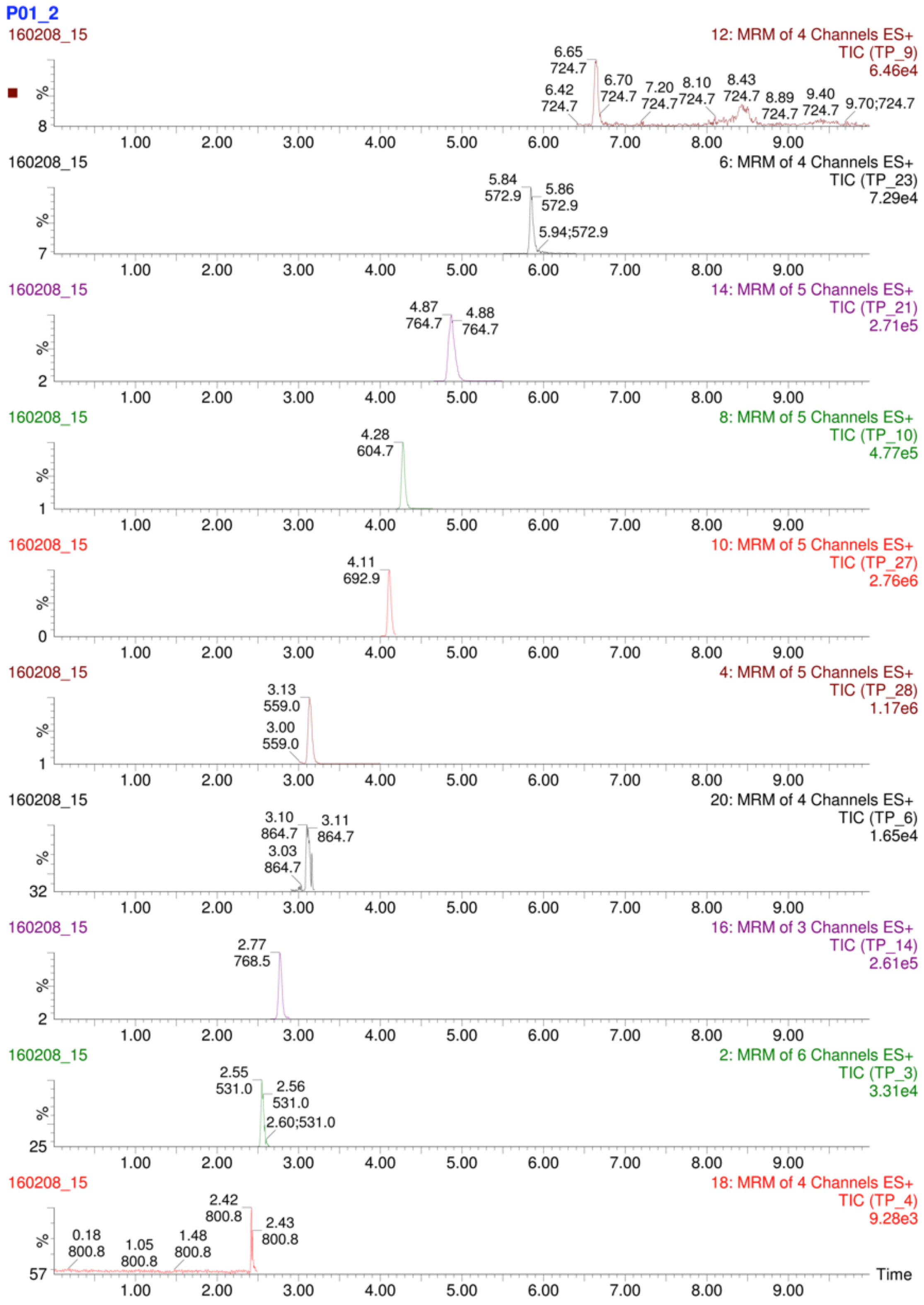

e) Gradient 3; endogenous (*T. pallidum*) PTP view

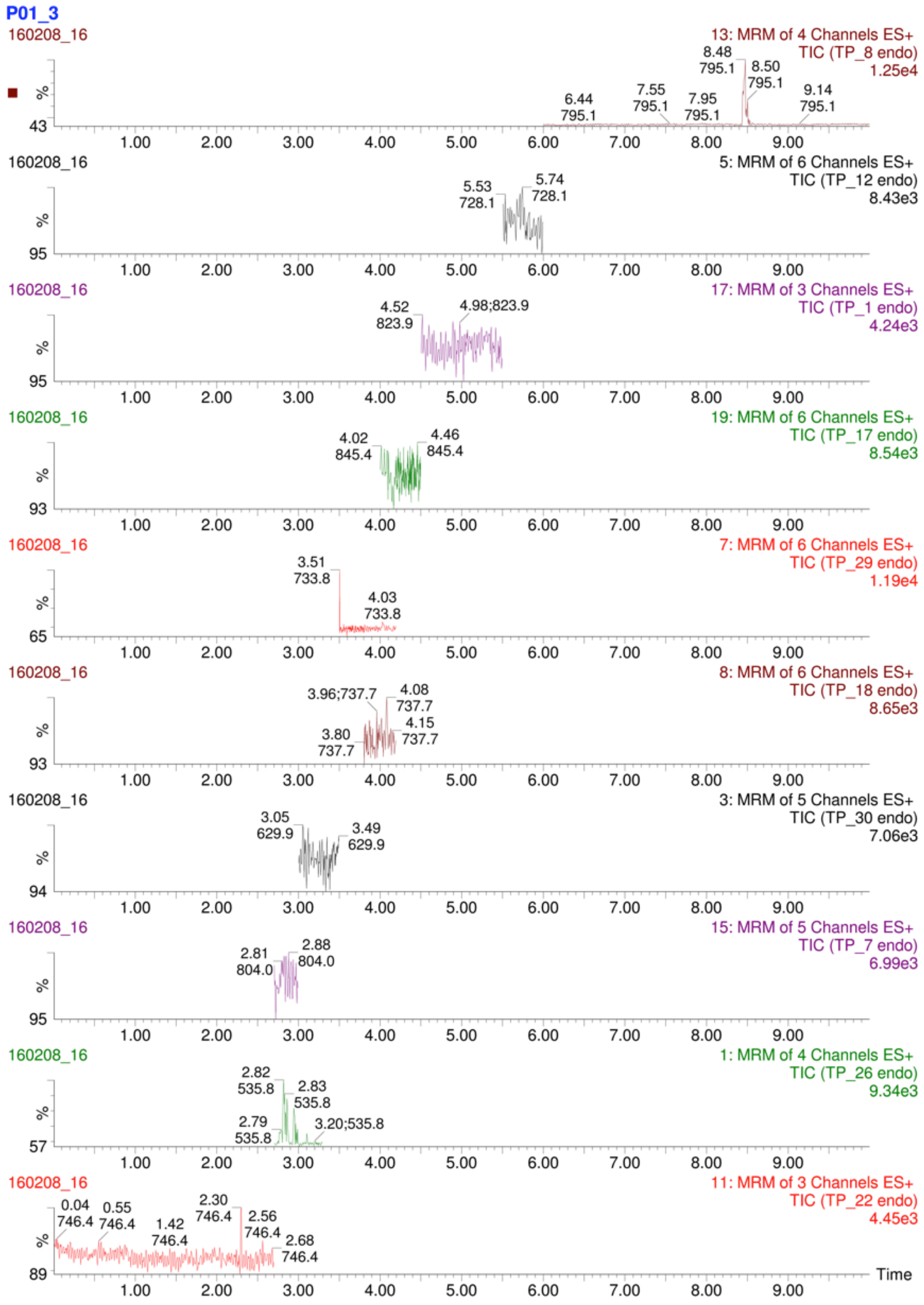

f) Gradient 3; hPTP view

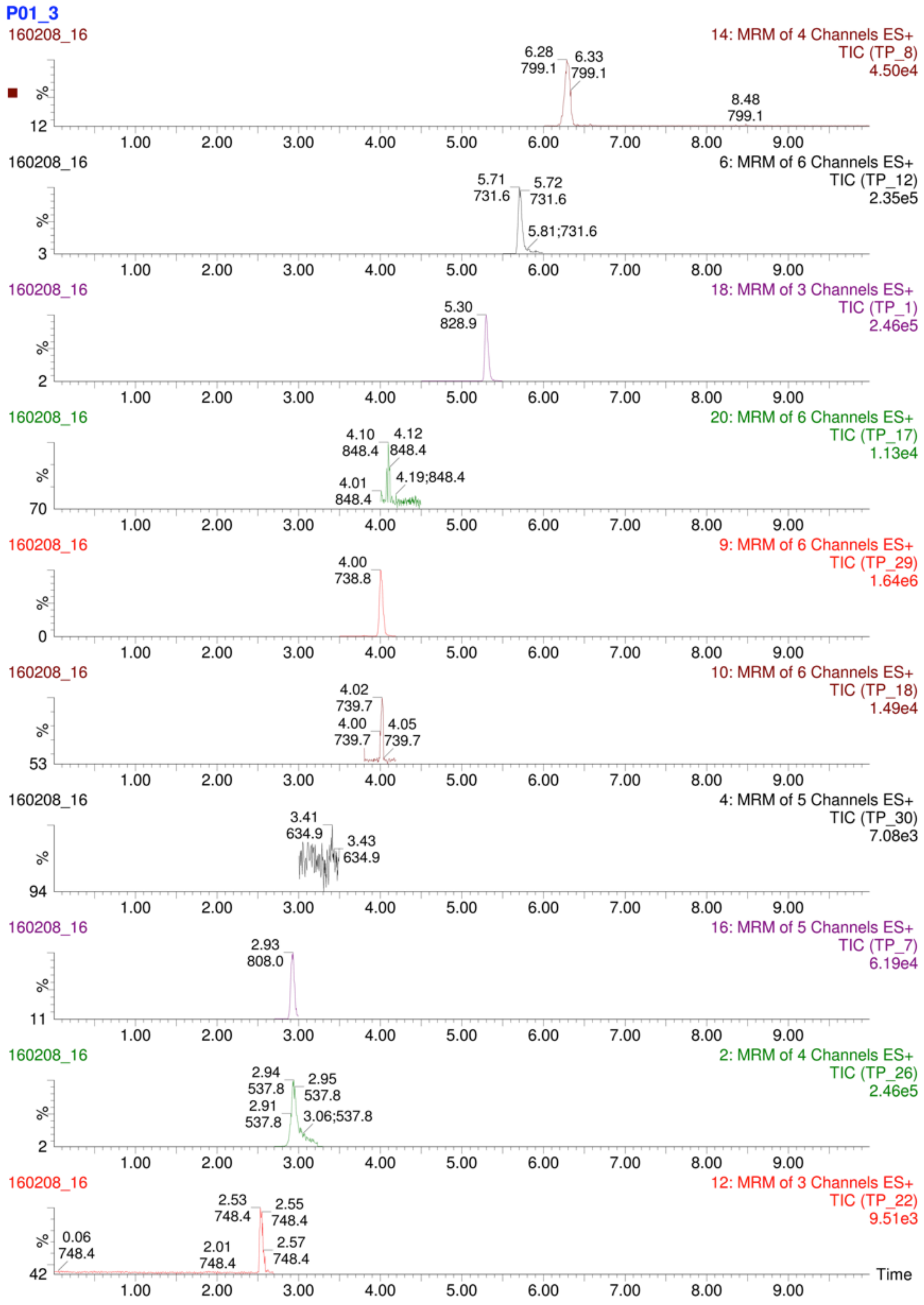

Supplement: Supplementary file 2 [file f1000research-7-18062-s0001.tgz › 805564f4-2b32-4450-af0c-167060a6ae7a.pdf]
