# Supplementary material for: Needle lost in the haystack: multiple reaction monitoring fails to detect Treponema pallidum candidate protein biomarkers in plasma and urine samples from individuals with syphilis
Source: F1000Res. 2018 Oct 9;7:336. Originally published 2018 Mar 19. [Version 2] doi: 10.12688/f1000research.13964.2 (PMC6248270; doi:10.12688/f1000research.13964.2)
Supplement: Peer review history

# Peer review: Referee response for version 2

**DOI:** 10.5256/f1000research.18062.r39229

**Published:** 2018-11-19

**Authors:** Mohd M. Khan

**Affiliations:** 1 University of Maryland, Baltimore, MD, USA

I have read this submission. I believe that I have an appropriate level of expertise to confirm that it is of an acceptable scientific standard.

# Peer review: Referee response for version 1

**DOI:** 10.5256/f1000research.15178.r35202

**Published:** 2018-08-22

**Authors:** Timothy Palzkill

**Affiliations:** 1 Department of Pharmacology and Chemical Biology, Baylor College of Medicine, Houston, TX, USA

This paper by Van Raemdonck describes the use of mass spectroscopy to identify T. pallidum proteins from plasma and urine from infected patients. If successful, such a method would be very useful in syphilis diagnostics, particularly with regard to reinfection. Thus, the work addresses a significant problem. However, although they could detect isotopically labeled peptides spiked into the samples, they could not detect T. pallidum proteins from the infecting organisms. Limit of detection experiments suggest this is due to the very low concentrations of T. pallidum proteins in plasma and serum samples. Thus, the main goal produced a negative result. However, there is considerable useful information in this study. The limit of detection experiments with T. pallidum bacteria that have been diluted and were unenriched or enriched with antibody beads provided interesting results on which proteins could be detected and how many bacteria per ml were needed for detection. In addition, the MRM experiments appear to be carefully designed and provide important limit of detection information for future studies. The discussion provides a useful assessment of limiting factors in the direct detection of T. pallidum antigen proteins.

 Comments:

 1.  Some of the description of LOD experiments with dilutions of T. pallidum on page 7, right column, paragraph 2, is difficult to follow.  The authors state “In total, eight unique T. pallidum proteins were found in the unenriched and enriched retentate…” but they do not state a dilution. Later in the paragraph, it states “Ten unique T. pallidum proteins were found in the highest concentration….” .  This is confusing. What condition does the eight unique proteins refer to?

 2. Page 7, right column, paragraph 2.  Tp47 is discussed twice in the paragraph with different gene names each time, ie, Tp47 (TP_0547) and Tp47 (TP_0574).

 3. Page 3, Introduction, paragraph 1, line 1. “T. pallidum, a non-culturable…”  Suggest updating based on recent publication in mBio on culturing T. pallidum.

 4. Figure 3. Please indicate the meaning of the asterisks on TP_0249, etc.

I have read this submission. I believe that I have an appropriate level of expertise to confirm that it is of an acceptable scientific standard.

# Response

**Published:** 2018-10-03

**Authors:** Kara Osbak

**Affiliations:** ErasmusMC, Belgium

1. **Some of the description of LOD experiments with dilutions of T. pallidum on page 7, right column, paragraph 2, is difficult to follow. The authors state “In total, eight unique T. pallidum proteins were found in the unenriched and enriched retentate…” but they do not state a dilution. Later in the paragraph, it states “Ten unique T. pallidum proteins were found in the highest concentration….” . This is confusing. What condition does the eight unique proteins refer to?**

 This ambiguous sentence has now been reworded- “In total, eight unique *T. pallidum* proteins were found in both the unenriched and enriched retentate dilution series in one or more of the concentrations analyzed”. This refers to the proteins that were commonly found in both experiments, regardless of concentration. The details of which are provided in Supplementary Table 3.

 The other sentence was also reworded for clarify “Ten unique *T. pallidum* proteins were found in the highest concentration (10 ^4^ bacteria/ml) four in the enriched retentate sample (N = 10) and non-enriched sample (N = 10), two proteins detected were unique to either the enriched or unenriched samples (Figure 3).”

**2. Page 7, right column, paragraph 2.  Tp47 is discussed twice in the paragraph with different gene names each time, ie, Tp47 (TP_0547) and Tp47 (TP_0574).**

 Thanks for pointing this out, this has been rectified to the actual ORF (TP_0574).

**3. Page 3, Introduction, paragraph 1, line 1. “T. pallidum, a non-culturable…”  Suggest updating based on recent publication in mBio on culturing T. pallidum.**

 Good idea, this reference has now been added.

**4. Figure 3. Please indicate the meaning of the asterisks on TP_0249, etc.**

 The asterisks refer to “*-proteins selected as candidate biomarkers in this study.”. This information has now been added

# Peer review: Referee response for version 1

**DOI:** 10.5256/f1000research.15178.r35906

**Published:** 2018-07-27

**Authors:** Mohd M. Khan

**Affiliations:** 1 University of Maryland, Baltimore, MD, USA

The article “Needle lost in the haystack: multiple reaction monitoring fails to detect *Treponema pallidum* candidate protein biomarkers in plasma and urine samples from individuals with syphilis” focuses on developing targeted proteomics based assay to detect and validate potential biomarkers of *Treponema pallidum *infection. The manuscript is well written, the rationale behind the work is justified, and the methods section is detailed. 

 I would suggest few points as following:  

- Please add the details about participant consent and study approval by the Institutional Review Board (IRB).
- Why only 4 urine samples were analyzed? Which protein marker(s) supposedly should be detected in urine? And which one(s) in plasma? Please consider adding the details and rationale.
-  Authors have used microflow for the experiments on quadrupole, which has lower sensitivity. Perhaps targeted experiments using a nanoflow setup, as was done for experiments using orbitrap, will get better sensitivity. 
- The synthetic peptides were added after SPE clean-up. Why weren't they added before SPE to determine losses?
-  Please supply more information on how LOD was calculated; it was based on the dilution series? The readers can use the explanation of calculations, if provided. 
- The spiking experiments should have been done in real matrix? 
  -  Please comment more on PTP selection? Are they unique? What about labile residues? Was anything done to look at the methionine oxidation?  Deamindations? etc.
- The synthetic peptides were 95% pure. Were they quantified? (AAA?)

 

 The paper makes an interesting list of its shortcomings in the discussion, which is helpful. A lot of the critique is already self-proclaimed.The overall conclusion of manuscript is: "A lot of effort and fine-tuning of sample prep/method development will be needed for biomarker discovery and validation." Biomarker validation is time-consuming and challenging, perhaps some orthogonal experiments should have been done (such as western blotting) to be able to know if the global data acquired using spectral-count was good enough before moving on to the MRM experiments. Nonetheless, authors have done a great job in discussing the shortcomings and in writing the paper.

I have read this submission. I believe that I have an appropriate level of expertise to confirm that it is of an acceptable scientific standard, however I have significant reservations, as outlined above.

# Response

**Published:** 2018-10-03

**Authors:** Kara Osbak

**Affiliations:** ErasmusMC, Belgium

**Please add the details about participant consent and study approval by the Institutional Review Board (IRB).**

The following information is available under the section “Consent and Ethics Approval”: *“The prospective observational cohort study (SeTPAT ClinicalTrials.gov # NCT02059525) that provided the clinical samples used in this study was approved by the Institutional Review Board of the Institute of Tropical Medicine Antwerp and the Ethics Committee of the University of Antwerp (13/44/426), Belgium. Written informed consent for publication of the participants’ anonymized details was obtained from the participants.”*

**Why only 4 urine samples were analyzed? which protein marker(s) supposedly should be detected in urine? and which one(s) in plasma? please consider adding the details and rationale.**

As this was an exploratory biomarker study we did not stratify our biomarker selection by biofluid type, thus the potential biomarkers mentioned in this study were theoretically applicable to urine and blood. No previous studies of this type have been performed, hence our selection, as described, was based on our shotgun proteomics studies of *Trepoenema pallidum* during rabbit infection, literature inferences on previous microarray studies and physiochemical characteristics that would be amendable to MRM detection.

Admittedly, only analyzing four urine samples is a small number. It became apparent that our experimental strategy was not working after analyzing the initial set of samples that we decided not to go further. Despite this small number, we believe this information might be useful for other groups considering employing similar methods.

**Authors have used microflow for the experiments on quadrupole, which has lower sensitivity. Perhaps targeted experiments using a nanoflow setup, as was done for experiments using orbitrap, will get better sensitivity.**

Indeed, one would expect higher sensitivity with the nanoflow set-up, however, to analyze larger volumes of patient material, which might also increase the sensitivity, the microflow set-up is more advantageous. Moreover, targeted microflow LC-MS/MS experiments offer the benefit of increased throughput (the initial goal was to develop method to analyze larger sample cohorts in a short time) and robustness.

**The synthetic peptides were added after SPE clean-up. Why weren't they added before SPE to determine losses?**

In this exploratory study, multiple candidate biomarkers were included in the targeted setup to evaluate their potency. Therefore, it was not yet clear in which final concentration these synthetic peptides should be spiked into the samples. At this stage of the study, the synthetic peptides were also not exactly quantified (i.e. AQUA Basic peptides), which would make the determination of losses during sample preparation not precise.

**Please supply more information on how LOD was calculated; it was based on the dilution series? The readers can use the explanation of calculations, if provided.**

The LOD calculations were based on the dilution series of *T. pallidum* in PBS, which were either enriched or unenriched with magnetic beads coupled with polyclonal antibodies directed against *T. pallidum*. These were then subjected to LTQ Orbitrap analyses. Two unique *T. pallidum* proteins, Cytoplasmic filament protein A (TP_0748) and Lipoprotein antigen Tp47 (TP_0547), were found in the 300 bacteria/ml fraction in the enriched and unenriched samples, respectively. Therefore, the LOD based on a high-resolution LTQ-Orbitrap instrument was approximately 300 bacteria/ml PBS for both the antibody enriched and unenriched samples, meaning there was no significant improvement in LOD using bead enrichment. These results are detailed in Supplementary File 3. Furthermore, rough concentration calculations based on previous studies were presented in Supplementary Table 4 which estimated that the concentration of *T. pallidum*/ target PTPs in human serum would be in the femtomoles per liter range during human *T. pallidum* infection (calculations presented in Supplementary File 4).

**The spiking experiments should have been done in real matrix?**

Indeed, the final dilution series of the labeled synthetic peptides, that would be used to determine the absolute concentration of the candidate biomarkers, would have been done in real matrix. However, at this point of the study the goal was to evaluate the abundance of the selected proteotypic peptides before using absolutely quantified labeled peptides (e.g. AQUA Ultimate). Therefore, it was decided to tune and optimize the LC-MS/MS parameters of each labeled peptide without any matrix to determine the most optimal instrument settings.

**Please comment more on PTP selection? Are they unique? What about labile residues? Was anything done to look at the methionine oxidation?  deamindations? etc**

Due to the lack of available MS datasets about the *Treponema pallidum* proteome (no library available), proteotypic peptides of each candidate protein biomarker were predicted *in silico*. As described, ESP predictor (Fusaro et al, 2009 Nat. Biotechnology) was used to find the most suitable proteotypic peptides based on 550 physico-chemical parameters including potential modifications (e.g. oxidation of methionine, deamidation, phosphorylation etc.). Best scoring peptides were selected for each of the proteins. Moreover, the PTPs that were selected were subjected to BLAST analyses to confirm their uniqueness.

**The synthetic peptides were 95% pure. Were they quantified? (AAA?)**

During this exploratory study AQUA Basic peptides (Thermo Fisher Scientific) were used to evaluate the abundance of the selected proteotypic peptides. Although the quantity of the PTPs were specified in the leaflet, the peptides were purchased in a lyophilized formulation as one aliquot. Therefore, they are not suited as reference for absolute quantification. In a next step, AQUA Ultimate peptides (with a high concentration precision) would have been used to determine the absolute abundance of the protein biomarkers.
